# Impact of Artificial Intelligence–Based Technology on Nurse Management: A Systematic Review

**DOI:** 10.1155/2024/3537964

**Published:** 2024-10-12

**Authors:** Alberto Gonzalez-Garcia, Silvia Pérez-González, Carmen Benavides, Arrate Pinto-Carral, Enedina Quiroga-Sánchez, Pilar Marqués-Sánchez

**Affiliations:** ^1^Faculty of Health Sciences, Nursing and Physiotherapy Department, University of Leon, León 24007, Spain; ^2^Department of Electric, Systems and Automatic Engineering, SALBIS Research Group, University of Leon, León 24007, Spain; ^3^Faculty of Health Sciences, Nursing and Physiotherapy Department, SALBIS Research Group, Campus of Ponferrada, University of Leon, León 24402, Spain

## Abstract

**Aim:** To describe the use of artificial intelligence (AI) by nurse managers to enhance management, leadership, and healthcare outcomes.

**Background:** AI represents a significant transformation in healthcare management by enhancing decision-making, communication, and resource optimization. However, the integration and strategic application of AI in nursing management are underexplored, particularly regarding its impact on leadership roles and healthcare delivery.

**Methods:** Methodological guidelines described by PRISMA were followed, and quality was assessed using the Joanna Briggs Institute (JBI) methodology. The databases searched included the Web of Science, Scopus, CINAHLi, and PubMed. The review included quantitative, qualitative, and mixed-method studies published between January 2015 and April 2024.

**Results:** Fourteen studies were selected for the review. The key findings indicate that AI technologies facilitate better resource management, risk assessment, and decision-making. AI also supports nurse managers in leading changes, enhancing communication, and optimizing administrative tasks.

**Conclusion:** AI has been progressively integrated into nursing management, demonstrating significant benefits in operational efficiency, decision support, and leadership enhancement. However, challenges, such as resistance to technological change and ethical complexities, need to be addressed.

**Implications for Nursing Management:** Specific training programs for nurse managers are essential to optimize the integration of AI. Such programs should focus on the management of AI applications and data analyses. In addition, creating interdisciplinary groups involving nurse managers, AI developers, and nursing staff is crucial for tailoring AI solutions to meet the unique needs of healthcare settings.

## 1. Introduction

Artificial intelligence (AI) is a technological system of our time that is disruptively transforming human activity, offering an unlimited set of possibilities and perspectives [1–4]. The origin of AI can be traced back to the Dartmouth College conference, responding to the question posed by John McCarthy: “Can machines think?” [5]. Thus, AI can be defined as the simulation of human intelligence by machines designed to perform activities that were previously exclusive to human thought, such as decision-making, problem-solving, and complex activities, with the potential to match or exceed human capabilities [6–8]. This concept includes machine learning, natural language processing, environmental perception, information classification, and neural networks, among others [9].

For the purposes of this study, AI is defined as the simulation of human intelligence by machines that are capable of performing tasks requiring human cognitive functions, such as decision-making, problem-solving, and learning from experience [6].

The use of AI in healthcare is advancing rapidly and has had a significant impact on enhancing patient diagnostics, improving prevention and treatment, and the sustainability of organizations [10, 11]. It is also instrumental in the analysis of skin tumors and various other pathologies [12–14], as well as in facilitating equitable access to healthcare services [11, 15]. In the field of nursing, AI can reduce administrative work and improve continuity and evolution of care [16, 17]. In addition, AI has been used to classify patients according to their needs, assess risks of ischemic stroke and pressure ulcers, predict falls, enhance knowledge of urinary tract infections, and even predict the risk of loneliness and frailty [18–20].

The use of AI in administration has grown rapidly in recent years, transforming the way organizations operate and manage resources [10]. AI offers new tools to optimize workflows, reduce administrative burdens, and improve decision-making processes [8]. In the context of healthcare administration, AI has proven to be important for managing large datasets, automating routine tasks, and facilitating predictive analytics to anticipate resource needs and patient demands [21]. These applications not only improve operational efficiency but also enable managers to focus on higher level strategic tasks, ultimately leading to better organizational outcomes.

Nurse managers occupy a key position due to their responsibilities in leadership, resource management, planning, and decision-making [22]. They face numerous challenges, such as work overload, need for quick decision-making, and efficient resource management. These challenges can be effectively addressed by applying AI [23]. AI has become an essential component in the performance of management functions, providing tools that reduce administrative burdens and facilitating data-driven decision-making [23–25]. Therefore, nurse managers need not only to develop technological competencies but also to adopt a strategic vision to lead this technological change, which is aimed at improving patient care [8, 26, 27]. In addition, their role as change leaders is crucial for the effective integration of AI into nursing teams [28].

Therefore, in the field of AI, it is crucial to explore how technologies such as machine learning, including deep learning and natural language processing, create new opportunities for nurse managers [8, 29, 30], and more importantly, we must analyze how nurse managers need to redefine their roles [8, 31]. Hence, we posed the question of how nurse managers utilize AI to enhance management, leadership, and healthcare outcomes. By addressing this question, we aimed to build a comprehensive body of knowledge that advances the understanding and application of AI in the healthcare sector.

To address this research question, the objective of this study was to describe how nurse managers utilize AI.

## 2. Materials and Methods

### 2.1. Design

This study adopted a systematic review methodology, aligned with the Joanna Briggs Institute (JBI) methodology for systematic reviews and adhering to the guidelines established by the Preferred Reporting Items for Systematic Reviews and Meta-Analyses (PRISMA) [32–34]. The research question was formulated using the population-concept-context (PCC) framework [35]. It was stated as follows: How is AI (C) used in nurse management (P) to improve leadership, decision-making, planning, and resource management [C]? The review protocol was registered in PROSPERO CRD42024534057.

### 2.2. Search Strategy

The search for articles was conducted in April 2024 using the Cochrane Library and PROSPERO databases to identify previous reviews related to nurse management and AI. No published or ongoing review has been published to date. The search strategy involved three steps: (1) an initial search was performed to identify all keywords related to nurse management and AI; (2) a comprehensive search through the Web of Science, Scopus, CINAHL, and PubMed databases was conducted using the identified keywords; and (3) references in the bibliographies of the identified articles were searched to identify additional sources. The keywords used in the databases included “nurse manager,” “nurse supervisor,” “nursing program manager,” “nurse unit manager,” “chief nurse executive,” “nurse administrator,” “director of nursing,” “head nurse,” “frontline manager,” “nursing director,” “nursing executive,” “AI,” and “AI,” as detailed in Table 1.

### 2.3. Inclusion and Exclusion Criteria

For this systematic review, original articles employing quantitative, qualitative, and mixed methods that addressed both nurse manager roles and AI were included. These articles must have been published in peer-reviewed journals between January 1, 2015, and April 2024. The decision to limit the search to articles published from 2015 onwards was based on the rapid evolution of AI technologies in healthcare and the relatively recent application of these technologies in nurse management. Given that AI-based technologies have developed substantially in the past decade, studies prior to 2015 may not accurately reflect the current state of AI's integration into nursing management practices. In addition, no language filters were applied in the search to avoid excluding potentially relevant studies from non-English sources. This decision was made to capture a broader range of research, considering the scarcity of studies in this specific field of nurse management and AI.

Exclusion criteria encompassed articles that did not specifically refer “nurse manager” or “AI.” Reviews, thesis papers, and non–peer-reviewed articles were excluded from the review.

### 2.4. Definition of Nurse Manager

For the purposes of this research, a “nurse manager” is defined as an individual responsible for translating an organization's strategy, managing resources, coordinating nursing care, leading nursing teams, planning, implementing innovative practices, and contributing to the evaluation of the services provided [36].

### 2.5. Study Selection and Outcome

**Figure 1:**
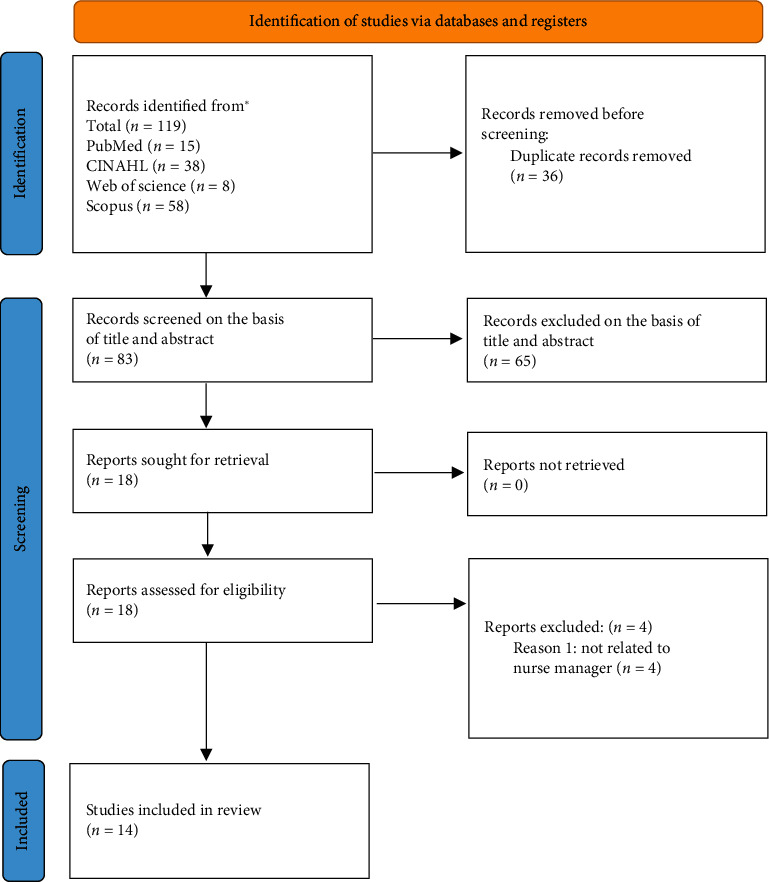
PRISMA flowchart.

A total of 119 articles were identified in the selected databases. No additional articles were identified through manual search. Articles were managed using the Mendeley bibliography management tool, in which 83 duplicates were identified and subsequently removed. The titles and abstracts of the remaining articles were independently reviewed by AG and SP. Eighteen full-text articles that met the inclusion criteria were further reviewed by AG and EQ. During the full-text review, three articles were excluded because they did not meet the inclusion criteria. To resolve discrepancies regarding the inclusion of two articles, AG and EQ met with PM on two occasions, using the consensus method to reach an agreement on their inclusion. Finally, 14 articles satisfied all criteria and were included in the final review. The selection process is depicted in the PRISMA flowchart in Figure 1.

### 2.6. Quality Appraisal

The quality assessment of the articles was independently conducted by AP and CB using two JBI assessment tools. The first tool assessed quantitative research articles, while the second tool, the JBI Qualitative Checklist, evaluated their respective types of studies [37, 38]. These JBI checklists are validated and widely used instruments that allow for quality evaluations to be categorized as “yes,” “no,” “uncertain,” or “not applicable” across various types of research. Any discrepancies between the reviewers' assessments were resolved by discussion.

In addition, the overall methodological quality of this systematic review was assessed using the AMSTAR 2 tool (A MeaSurement Tool to Assess systematic Reviews) [39]. This tool is designed to evaluate systematic reviews that include randomized or nonrandomized studies. AMSTAR 2 consists of 16 items that assess various aspects of methodological quality, such as the clarity of the research question, the completeness of the literature search, the justification for study selection, and the assessment of the risk of bias in the included studies, among others.

The risk of bias in individual studies was assessed at the study level using JBI critical appraisal tools for both quantitative and qualitative studies. The risk of bias informed the narrative synthesis, categorizing studies as low, moderate, or high risk, and guiding the weighting of evidence. In addition, a qualitative sensitivity analysis was conducted by excluding high-risk studies to assess the potential impact on the review's conclusions.

### 2.7. Data Extraction

Data relevant to the review question were independently extracted by two investigators AG and EQ using specifically developed forms that were agreed upon by all members of the research team. The extracted data are presented in Supporting Information 1 Tables S1–S9 and served as the foundation for the subsequent data synthesis.

### 2.8. Data Analysis and Synthesis

Given the heterogeneity of the data, meta-analysis was not feasible. Instead, significant elements from the studies were meticulously examined, including (1) author and year of publication, (2) study design, (3) variables, (4) type of AI, (5) application of AI by the nurse manager, and (6) measurement of article quality. To meet the specific needs of this study, we employed both SWiM methodology and thematic synthesis [40]. The SWiM approach was used to summarize and group studies with similar designs and objectives, focusing on key study characteristics such as results, methodological quality, and variables related to the impact of AI on nurse management. This approach involved several key steps: (1) summarizing articles, results, and methodological quality; (2) identifying similar studies for comparison and grouping; (3) determining data relevant to the study objectives; and (4) synthesizing the available evidence in a coherent manner to yield results aligned with the research objectives.

After grouping the studies using SWiM, a thematic analysis was conducted to explore more in-depth patterns and themes within the studies. This analysis helped identify, code, and categorize the main themes that emerged from the data. Themes were identified by reading through the included studies multiple times and highlighting recurring concepts and patterns related to the impact of AI on nurse management. These themes were then coded and grouped into broader categories, such as decision-making, resource management, leadership enhancement, and communication optimization.

The coding system used to extract and categorize these themes was developed and registered by AG. Specifically, the intellectual property of this characteristic extraction process was registered with the Spanish Ministry of Culture under number 00/2024/3056. The thematic analysis process was carried out independently by AG and EQ, who compared their coding results to ensure consistency. No significant discrepancies were found during the coding process, and therefore no additional meetings were required to resolve differences. The themes were validated by cross-referencing them with the primary findings of the included studies to ensure they accurately represented the data. This thematic approach allowed for a clear synthesis of the qualitative findings, providing a structured interpretation of the literature.

## 3. Results

This review identified 119 studies from database searches. Eighteen articles were selected after reviewing their titles and abstracts, of which 14 were included in the review (Figure 1). Of these, six were quantitative [24, 41–45] and eight were qualitative [1, 6, 46–51] (see Table S1, Supporting Information 1).

However, it should be noted that a significant portion of the current evidence on nurse management and AI comprises articles that perform conceptual analyses or discussion focusing on the debate on the potential for transforming management practices (see Table S2, Supporting Information 1).

### 3.1. Quality Analysis

The results of the quality analysis varied from moderate to low. Quantitative studies generally exhibited moderate adherence to the quality criteria, with scores ranging between 50% and 75%. These scores indicate a reliable level of methodological consistency (see Table S3, Supporting Information 1).

In contrast, qualitative studies demonstrated a greater variation in quality scores, ranging from 10% to 80% (see Table S4, Supporting Information 1). Lower scores, predominantly found in the conceptual analyses, suggest potential discrepancies with the JBI qualitative assessment tool. Nevertheless, the inclusion of these studies was deemed essential for broadening our understanding.

**Figure 2:**
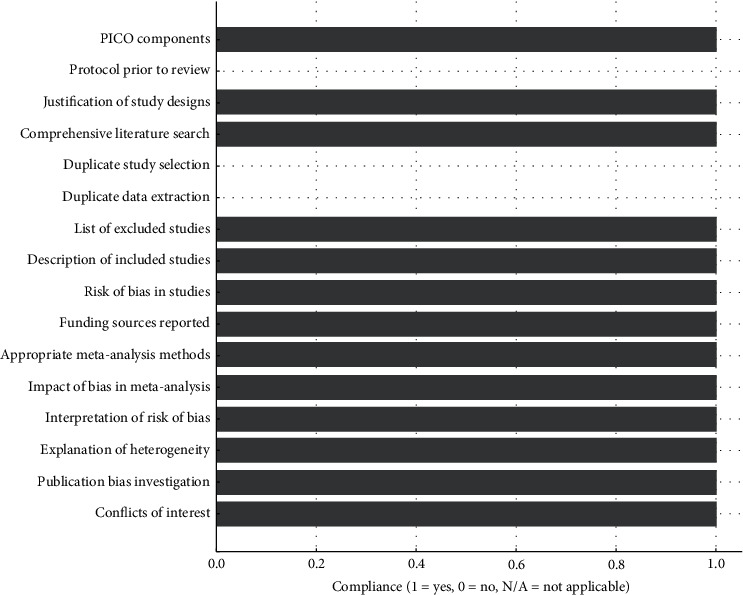
AMSTAR 2 quality assessment results.

The AMSTAR 2 assessment showed high compliance with the evaluated items, indicating that the review follows a sound methodological approach, ensuring the integrity and validity of the results (Figure 2). Only the items referring to the performance of meta-analysis were marked as not applicable due to the nature of the data and the methodology used.

The categorization of studies based on their risk of bias (low, moderate, and high) guided the weighting of evidence in the narrative synthesis. In our research, 14% of the studies were categorized as low risk, 79% as moderate risk, and 7% as high risk. This distribution highlights the predominance of studies with moderate risk and underscores the need for careful interpretation of findings from high-risk studies (see Table S5, Supporting Information 1).

As for the qualitative sensitivity analysis to assess the robustness of the results, the overall conclusions were reassessed by excluding studies identified as being at high risk of bias. The study identified as high risk was by Ergin et al. [44], which was excluded from the sensitivity analysis. The exclusion of the high-risk study did not significantly alter the overall results, suggesting the reliability of the review's conclusions. Although the conclusions were more robust based on studies with low and moderate risk of bias, no articles were excluded to obtain a greater body of knowledge, even though this introduced certain uncertainties.

### 3.2. Applicability to Key Nursing Management Areas

One of the major concerns today is the usefulness of AI in key areas, such as decision-making, material resource management, human resource management, communication, values and ethics, team management, and conflict management. AI integration enhances decision-making processes by providing real-time data and predictive insights, which are crucial for strategic planning and responses in nursing settings. For example, Blouin [48] and Chang, Jen, and Su [24] highlighted AI's role of AI in improving the decision-making capabilities. Han et al. [41] and Li et al. [45] demonstrated how AI supports leadership qualities by facilitating innovative and effective management practices adapted to dynamic healthcare environments.

In terms of communication, Laukka et al. [1] and Clancy [47] illustrated how AI improves coordination and information sharing among nursing teams.

Although the impact of AI on ethics and values has been variably addressed, studies such as that by Huang et al. [43] emphasize the importance of ethical AI use in management.

However, conflict management and integrated team management have been addressed less frequently in the literature. This gap underscores the need for further research on how AI can be leveraged in conflict resolution and teamwork (see Table S6, Supporting Information 1).

### 3.3. Strengths, Weaknesses, Opportunities, and Threats (SWOT) Analysis of IA in Nursing Management

The integration of AI into nursing management has marked a radical transformation in the execution of management roles. Consequently, it is essential to conduct a thorough analysis of the literature to assess the SWOT associated with incorporating AI. This analysis provides a comprehensive understanding of the potential impacts and strategic considerations necessary for effectively leveraging AI in this field (see Table 2).

The importance of AI in data-driven decision-making and innovation is clearly evidenced by the SWOT analysis. In addition, the automation of administrative tasks increases the efficiency of nursing management activities, allowing nurses to focus on individual patient care. However, the analysis also identifies threats and weaknesses that require attention, such as the need for improved training, which can hinder effective development and implementation. Moreover, overreliance on technology can lead to a decline in essential person-to-person communication skills. Ethical and privacy concerns are crucial, especially in the context of data management.

### 3.4. Integration of AI in Nursing Management Practice

The integration of AI into nursing management has significantly influenced various practical aspects. Blouin [48] and Huang et al. [43] highlighted its role in managing staff shortages and in improving recruitment, retention, and job satisfaction while also reducing administrative burdens. Further research has indicated that technologically advanced healthcare environments enhance the quality, safety, and communication, as demonstrated by Chang et al. [24] and Han et al. [41], Ergin et al. [44], and Huang et al. [43] (see Table S7, Supporting Information 1).

### 3.5. AI Barriers and Challenges

The presence of AI in the nurse manager environment presents various challenges and barriers, as highlighted in numerous studies. These challenges include resistance to technological change, ethical and structural complexities, and high costs associated with new technologies, as noted by Blouin [48]. Han et al. [41] and Ergin et al. [44] emphasized the need for significant cultural changes within healthcare settings to accommodate AI-driven processes. Research conducted by Chang et al. [24] and Chen et al. [49] discussed logistical challenges in aligning AI applications with existing nursing management priorities, underscoring the importance of enhanced AI training and improved collaboration between developers and healthcare professionals. In addition, Huang et al. [43] and Laukka et al. [1] pointed out the limitations of AI in sensitive areas such as patient surveillance, ethical data management, and privacy concerns (see Table S8, Supporting Information 1).

### 3.6. Future Trends and Recommendations for the Integration of AI in Nursing Management

From the articles analyzed, it is evident that AI will be progressively and continuously integrated into nursing management, underscoring the need for its strategic and reflective use to fully harness the transformative potential of practice management. Studies indicate that AI will help mitigate resource shortages, improve team workflows [6], and enhance the well-being of nurses [41]. Recommendations call for robust training and significant development of ethical principles [43, 44], along with the involvement of nurse managers in developing AI strategies [1, 45] (see Table S9, Supporting Information 1).

## 4. Discussion

This review aimed to describe nurse managers' use of AI, which is integrated into key areas, such as leadership, decision-making, communication, and conflict management. The findings of this systematic review highlight the current state of research on the application of AI in nurse management. Our results indicate that while there is a growing interest in leveraging AI for enhancing nursing management practices, the evidence base remains varied in quality and scope. Studies with low to moderate risk of bias provide insights into how AI can be used to improve decision-making, resource management, and overall efficiency in nursing management. For example, studies by Han et al. [41] and Chang et al. [24] demonstrated robust methodologies and contributed valuable data on the practical applications of AI. These findings are consistent with other reviews in the field that emphasize the potential of AI to transform healthcare management [52, 53]. However, the predominance of studies with moderate risk of bias and the inclusion of conceptual analyses without empirical data suggest that more rigorous empirical research is needed. This aligns with the observations of previous studies that have called for higher quality studies to substantiate the theoretical benefits of AI in healthcare settings [54, 55].

AI contributes to decision-making by providing real-time data and predictive analytics, which are essential for planning management strategies. Furthermore, AI not only enhances performance efficiency but also transforms data management by enabling faster and more accurate access to critical information [56]. The results of the review clearly demonstrate how AI facilitates innovation and people-centered leadership practices, reinforcing the strategic change processes necessary in continuously evolving healthcare organizations [21, 57]. In terms of communication, AI improves coordination and information dissemination. However, it is necessary to adapt the structure of healthcare organizations, processes, and training to maximize efficiency and avoid interruptions in communication [58, 59]. Regarding ethical considerations, this review emphasizes the need to deepen our understanding of the impact of AI on ethics. Jobin et al. [60] and Mittelstadt et al. [61] argued that there is a crucial need to enhance knowledge of how to resolve ethical dilemmas related to privacy, autonomy, and responsibility.

The SWOT analysis highlights the strengths of AI in improving administrative efficiency and decision-making. In particular, AI-supported decision-making based on real-time and predictive data accelerates responses to problems, thereby reducing the time between problem identification and resolution [62, 63]. The analysis also highlights significant threats such as the loss of essential communication skills due to technological dependence. Similarly, several studies have highlighted privacy concerns in data management, pointing to privacy breaches and loss of trust in communications mediated by AI-based technology [61, 64, 65].

In terms of challenges and barriers, our findings indicate that resistance to change is one of the main obstacles, along with ethical complexities and difficulty in integrating these technologies with electronic health records. One possible explanation for resistance to change could be concerns about employment and its transformation [66]. In this regard, various studies suggest that knowledge management will help transform healthcare organizations by advancing AI, which, as our findings show, points to data security as one of the main ethical barriers [67, 68].

Finally, the review highlights the progressive integration of AI in nurse management and emphasizes the need for a strategic approach to address this process, carefully assessing how it responds to the needs of nurse managers. Coordinated actions with legislators, policymakers, and researchers facilitate the integration and adoption of these new technologies [69, 70].

This systematic review has several limitations. First, AI research related to nurse management is a practice that is developing rapidly; however, it is still supported by limited evidence. Despite recognizing the positive outcomes of current uses and the direction of conceptual analyses, a significant gap remains in the research. Second, the high heterogeneity of the data limits the possibility of conducting a meta-analysis. Finally, it should be noted that although this review contributes to building a body of knowledge, further research in the key areas of nurse management is needed to generalize the evidence emerging from this study.

This research shows there is a need for further empirical studies investigating the impact of AI on nursing management in key areas such as leadership, decision-making, predictive analytics, conflict management, teamwork, time management, and others. In addition, the body of knowledge from this research could guide future studies on how to develop simulation scenarios where nurse managers can train key competencies using AI in safe environments.

## 5. Conclusions

This review provides a comprehensive understanding of the growing integration of AI into nurse management practices. The findings underscore the significant role AI plays in enhancing decision-making, improving communication, optimizing resources, and supporting data management within healthcare organizations. AI has proven to be a transformative tool, enabling nurse managers to streamline processes and improve both patient care and operational efficiency.

However, despite these clear advantages, the adoption of AI is not without challenges. Nurse managers must navigate barriers such as resistance to technological change, ethical concerns surrounding data privacy and autonomy, and the complex task of integrating AI systems with existing electronic health records. These challenges call for targeted strategies in change management, continuous education and training for nurse managers, and the development of policies that ensure ethical data use and transparency.

To maximize the potential of AI in nursing management, it is crucial to create interdisciplinary collaborations between healthcare professionals and AI developers, ensuring that AI solutions are tailored to the unique needs of nursing leadership. In addition, investing in robust training programs will equip nurse managers with the necessary skills to effectively utilize AI technologies, ultimately enhancing patient outcomes and organizational performance.

AI represents a powerful tool that can significantly enhance the management of healthcare organizations. For nurse managers in particular, AI has the potential to improve both efficiency and financial sustainability by optimizing key processes such as workforce management, resource allocation, and communication. Moreover, AI supports strategic decision-making and leadership, allowing nurse managers to better anticipate changes, manage conflicts, and enhance overall organizational performance. By leveraging AI, nurse managers can streamline operations and focus on driving improvements in patient care and operational outcomes, making it an indispensable component of modern, sustainable healthcare management.

### 5.1. Implications for Nurse Management

The integration of AI could bring about a disruptive change in the way nurse managers perform their functions. It has the ability to improve efficiency in decision-making, communication, process redesign, and optimization of staff workflows, while also decreasing workloads and improving the work climate of the organization. In addition, it could be used as a predictive tool in sensitive areas, such as recruitment and retention of personnel and anticipation of peaks in demand, allowing the optimization of nursing staff. Therefore, nurse managers must develop advanced technological competencies to ensure ethical implementation of AI. However, AI implementation requires active participation in the development of applications to ensure adequate ethical and usability levels.

To optimize the integration of AI in nursing management, it would be appropriate to develop specific training programs that are oriented toward specific techniques in the management of AI and its applications in healthcare settings, such as data analysis and interpretation of AI algorithms. It is also essential to raise awareness among all staff about the potential benefits and challenges of AI to reduce resistance to change and promote a culture of innovation. In addition, the formation of interdisciplinary groups that include nurse managers, AI developers, and nurses to codesign solutions tailored to the specific realities of each healthcare organization would be appropriate.

## Figures and Tables

**Table 1:** Article search syntax.

**Database**	**Syntax**
CINAHL	(MH “artificial intelligence” OR TI artificial intelligence OR AB artificial intelligence) AND (MH “nursing management” OR MH “nurse administrators” OR TI nurse manager⁣^∗^ OR AB nurse manager⁣^∗^ OR TI nursing director⁣^∗^ OR AB nursing director⁣^∗^ OR TI nursing supervisor⁣^∗^ OR AB nursing supervisor⁣^∗^ OR TI “chief nursing officer” OR AB “chief nursing officer” OR TI “nursing executive” OR AB “nursing executive” OR TI “nursing leadership” OR AB “nursing leadership”)

Web of Science	TI = (“IA” OR “AI” OR “artificial intelligen⁣^∗^”) AND TI = (“nurs⁣^∗^ manage⁣^∗^” OR “nurse supervisor” OR “nursing program manager” OR “nurse unit manager” OR “chief nurse executive” OR “nurs⁣^∗^ administrat⁣^∗^” OR “director of nurs⁣^∗^” OR “head nurs⁣^∗^” OR “frontline manager” OR “nurs⁣^∗^ director” OR “nurs⁣^∗^ executiv⁣^∗^”) AND TS = (“head nurs⁣^∗^” OR “frontline manager” OR “nurs⁣^∗^ director” OR “nurs⁣^∗^ manag⁣^∗^” OR “first line nurs⁣^∗^ manage⁣^∗^” OR “nurse supervisor” OR “nursing program manager” OR “nurse unit manager” OR “chief nurse executive” OR “nurs⁣^∗^ administrat⁣^∗^” OR “director of nurs⁣^∗^” OR “nurs⁣^∗^ executiv⁣^∗^”) AND TS = (“IA” OR “AI” OR “artificial intelligen⁣^∗^”)

Scopus	TITLE-ABS-KEY (“nurs⁣^∗^ manage⁣^∗^” OR “nurse supervisor” OR “nursing program manager” OR “nurse unit manager” OR “chief nurse executive” OR “nurs⁣^∗^ administrat⁣^∗^” OR “director of nurs⁣^∗^” OR “head nurs⁣^∗^” OR “frontline manager” OR “nurs⁣^∗^ director” OR “nurs⁣^∗^ executiv⁣^∗^”) AND TITLE-ABS-KEY (“IA” OR “AI” OR “artificial intelligen⁣^∗^”)

PubMed	(“IA” OR “AI” OR “artificial intelligen⁣^∗^”) AND (“nurse Administrators” [Majr] OR “nursing, Supervisory” [Majr])

*Note:* The asterisk (^∗^) in the search syntax acts as a “wildcard” or truncation. This means that the database will include all words that share the same root preceding the asterisk. For example, “nurs” will include terms like “nurse,” “nursing,” “nurses,” etc. This helps to broaden the search to capture all variations of a keyword. It is an effective strategy to ensure that relevant results are not missed due to variations in terminology.

**Table 2:** SWOT analysis of AI in nursing management.

**Strengths**	**Weaknesses**
Improved decision making: AI provides tools to support data-driven decision-making, facilitating more accurate diagnoses and personalized treatments.	Lack of preparation and training: the absence of specific AI training for nursing staff may limit its effective use and integration into daily practice.
Operational efficiency: automation of administrative and routine tasks allows nursing staff to focus on direct patient care.	Technological dependence: overreliance on AI systems may diminish the clinical skills and independent judgment of the nursing staff.
Support for research and EBP: AI can speed up the translation of research into clinical practice, narrowing the gap between knowledge discovery and its application.	

**Opportunities**	**Threats**

Innovation in healthcare: the integration of AI technologies opens new avenues for innovation in nursing procedures and treatments.	Ethical and privacy concerns: managing large volumes of sensitive patient data raises concerns about privacy and consent.
Professional development and training: the adoption of AI in nursing practice encourages the need for continuous training, contributing to the professional development of staff.	Resistance to change: the implementation of new technologies may meet resistance from both nursing staff and patients.
Improved patient and staff satisfaction: by reducing workload and improving care precision, AI has the potential to increase satisfaction for both patients and nursing staff.	Implementation cost: the acquisition, maintenance, and updating of AI-based systems represent a significant cost for healthcare institutions.

## Data Availability

All data obtained from the analysis of the articles included in this review will be provided upon request by the corresponding author.

## Supporting Information

Supporting Information Supporting Information File 1: Table S1: article search syntax; Table S2: overview of conceptual synthesis and discussion; Table S3: quality appraisal of quantitative papers; Table S4: quality appraisal of qualitative papers; Table S5: risk of bias assessment for included studies; Table S6: applicability study of articles to key nursing management areas; Table S7: integration of AI in nursing management practice; Table S8: AI barriers and challenges; Table S9: future trends and recommendations for the integration of AI in nursing management.

## References

[1] Laukka E., Hammarén M., Kanste O. (2022). Nurse Leaders’ and Digital Service Developers’ Perceptions of the Future Role of Artificial Intelligence in Specialized Medical Care: An Interview Study. *Journal of Nursing Management*.

[2] Hajkowicz S., Sanderson C., Karimi S., Bratanova A., Naughtin C. (2023). Artificial Intelligence Adoption in the Physical Sciences, Natural Sciences, Life Sciences, Social Sciences and the Arts and Humanities: A Bibliometric Analysis of Research Publications From 1960–2021. *Technology in Society*.

[3] Wahl B., Cossy-Gantner A., Germann S., Schwalbe N. R. (2018). Artificial Intelligence (AI) and Global Health: How Can AI Contribute to Health in Resource-Poor Settings?. *BMJ Global Health*.

[4] Hoffmann C. H. (2022). Is AI Intelligent? An Assessment of Artificial Intelligence, 70 Years After Turing. *Technology in Society*.

[5] McCarthy J. (1956). *What Is Artificial Intelligence?*.

[6] Clancy T. R. (2020). Artificial Intelligence and Nursing: The Future Is Now. *The Journal of Nursing Administration: The Journal of Nursing Administration*.

[7] Krittanawong C., Zhang H., Wang Z., Aydar M., Kitai T. (2017). Artificial Intelligence in Precision Cardiovascular Medicine. *Journal of the American College of Cardiology*.

[8] Davenport T., Kalakota R. (2019). The Potential for Artificial Intelligence in Healthcare. *Future Healthcare Journal*.

[9] Martinez-Ortigosa A., Martinez-Granados A., Gil-Hernández E., Rodriguez-Arrastia M., Ropero-Padilla C., Roman P. (2023). Applications of Artificial Intelligence in Nursing Care: A Systematic Review. *Journal of Nursing Management*.

[10] Sunarti S., Fadzlul Rahman F., Naufal M., Risky M., Febriyanto K., Masnina R. (2021). Artificial Intelligence in Healthcare: Opportunities and Risk for Future. *Gaceta Sanitaria*.

[11] Zahlan A., Ranjan R. P., Hayes D. (2023). Artificial Intelligence Innovation in Healthcare: Literature Review, Exploratory Analysis, and Future Research. *Technology in Society*.

[12] Fakhoury M. (2019). Artificial Intelligence in Psychiatry. *Advances in Experimental Medicine and Biology*.

[13] French J., Chen C., Henson K. (2019). Identification of Patient Prescribing Predicting Cancer Diagnosis Using Boosted Decision Trees. *Lecture Notes in Computer Science*.

[14] Zhou X. Y., Guo Y., Shen M., Yang G. Z. (2020). Application of Artificial Intelligence in Surgery. *Frontiers of Medicine*.

[15] Bhatia R. (2021). Telehealth and COVID-19: Using Technology to Accelerate the Curve on Access and Quality Healthcare for Citizens in India. *Technology in Society*.

[16] Hwang G. J., Chang P. Y., Tseng W. Y., Chou C. A., Wu C. H., Tu Y. F. (2022). Research Trends in Artificial Intelligence-Associated Nursing Activities Based on a Review of Academic Studies Published from 2001 to 2020. *CIN: Computers, Informatics, Nursing*.

[17] Seibert K., Domhoff D., Bruch D. (2021). Application Scenarios for Artificial Intelligence in Nursing Care: Rapid Review. *Journal of Medical Internet Research*.

[18] Jeon E., Kim Y., Park H., Park R. W., Shin H., Park H. A. (2020). Analysis of Adverse Drug Reactions Identified in Nursing Notes Using Reinforcement Learning. *Healthc Inform Res*.

[19] An R., Chang G., Fan Y., Ji L., Wang X., Hong S. (2021). Machine Learning‐Based Patient Classification System for Adult Patients in Intensive Care Units: A Cross‐sectional Study. *Journal of Nursing Management*.

[20] O’Connor S., Yan Y., Thilo F. J. S., Felzmann H., Dowding D., Lee J. J. (2023). Artificial Intelligence in Nursing and Midwifery: A Systematic Review. *Journal of Clinical Nursing*.

[21] Mesko B., Hetenyi G., Gyorffy Z. (2018). Will Artificial Intelligence Solve the Human Resource Crisis in Healthcare?. *BMC Health Services Research*.

[22] Gunawan J., Aungsuroch Y. (2017). Managerial Competence of First-Line Nurse Managers: A Concept Analysis. *International Journal of Nursing Practice*.

[23] Rajkomar A., Dean J., Kohane I. (2019). Machine Learning in Medicine. *New England Journal of Medicine*.

[24] Chang C., Jen H., Su W. (2022). Trends in Artificial Intelligence in Nursing: Impacts on Nursing Management. *Journal of Nursing Management*.

[25] Elsayed W., Sleem W. (2021). Nurse Managers’ Perspectives and Attitude Toward Using Artificial Intelligence Technology in Health Settings. *Assiut Scientific Nursing Journal*.

[26] McGoldrick T. (2008). Leading Leaders. *Pa Nurse*.

[27] Sidey-Gibbons J. A., Sidey-Gibbons C. J. (2019). Machine Learning in Medicine: A Practical Introduction. *BMC Medical Research Methodology*.

[28] Robert N. (2019). How Artificial Intelligence Is Changing Nursing. *Nursing Management*.

[29] Harry A. (2023). The Future of Medicine: Harnessing the Power of AI for Revolutionizing Healthcare. *International Journal of Multidisciplinary Sciences and Arts*.

[30] Pettit R. W., Fullem R., Cheng C., Amos C. I. (2021). Artificial Intelligence, Machine Learning, and Deep Learning for Clinical Outcome Prediction. *Emerging Topics in Life Sciences*.

[31] U.S. Government Accountability Office (2022). Technology Assessment: Artificial Intelligence in Health Care: Benefits and Challenges of Machine Learning Technologies for Medical Diagnostics. https://www.gao.gov/products/gao-22-104629.

[32] Page M. J., McKenzie J. E., Bossuyt P. M. (2021). The PRISMA 2020 Statement: An Updated Guideline for Reporting Systematic Reviews. *PLoS Medicine*.

[33] Aromataris E., Munn Z. (2020). *JBI Manual for Evidence Synthesis*.

[34] Aromataris E., Fernandez R., Godfrey C., Holly C., Khalil H., Tungpunkom P. (2015). Summarizing Systematic Reviews: Methodological Development, Conduct and Reporting of an Umbrella Review Approach. *International Journal of Evidence-Based Healthcare*.

[35] Peters M. D. J., Godfrey C. M., Khalil H., McInerney P., Parker D., Soares C. B. (2015). Guidance for Conducting Systematic Scoping Reviews. *International Journal of Evidence-Based Healthcare*.

[36] González-García A., Pinto-Carral A., Pérez-González S., Marqués-Sánchez P. (2021). Nurse Managers’ Competencies: A Scoping Review. *Journal of Nursing Management*.

[37] Moola S., Munn Z., Tufanaru C., Aromataris E., Sears K., Sfetcu R. (2020). *Systematic Reviews of Etiology and Risk*.

[38] Lockwood C., Munn Z., Porritt K. (2015). Qualitative Research Synthesis. *International Journal of Evidence-Based Healthcare*.

[39] Shea B. J., Reeves B. C., Wells G. (2017). AMSTAR 2: A Critical Appraisal Tool for Systematic Reviews that Include Randomised or Non-Randomised Studies of Healthcare Interventions, or Both. *BMJ*.

[40] Campbell M., McKenzie J. E., Sowden A. (2020). Synthesis Without Meta-Analysis (SWiM) in Systematic Reviews: Reporting Guideline. *BMJ*.

[41] Han J., Kang H., Kwon G. H. (2020). Impact of Intelligent Healthscape Quality on Nurse Job Outcomes and Job Satisfaction: A Test of the Moderating Effect of Innovativeness. *Journal of Nursing Management*.

[42] Wang Y., Xie C., Liang C., Zhou P., Lu L. (2022). Association of Artificial Intelligence Use and the Retention of Elderly Caregivers: a Cross-Sectional Study Based on Empowerment Theory. *Journal of Nursing Management*.

[43] Huang K., Jiao Z., Cai Y., Zhong Z. (2022). Artificial Intelligence-Based Intelligent Surveillance for Reducing Nurses’ Working Hours in Nurse–Patient Interaction: A Two-Wave Study. *Journal of Nursing Management*.

[44] Ergin E., Karaarslan D., Şahan S., Çınar Yücel Ş. (2022). Artificial Intelligence and Robot Nurses: From Nurse Managers’ Perspective: A Descriptive Cross-Sectional Study. *Journal of Nursing Management*.

[45] Li X., Cheng M., Xu J. (2022). Leaders’ Innovation Expectation and Nurses’ Innovation Behaviour in Conjunction with Artificial Intelligence: The Chain Mediation of Job Control and Creative Self-Efficacy. *Journal of Nursing Management*.

[46] Fontenot J. (2024). Spotlight on Leadership. What Nurse Leaders Need to Know About Artificial Intelligence. *The Journal of Nursing Administration: The Journal of Nursing Administration*.

[47] Clancy T. R. (2020). Technology Solutions for Nurse Leaders. *Nursing Administration Quarterly*.

[48] Blouin A. S. (2023). Innovations in Nursing Workforce Management: Integrating Emerging Technologies With Proven Strategies. *The Journal of Nursing Administration: The Journal of Nursing Administration*.

[49] Chen Y., Moreira P., Liu W. W., Monachino M., Nguyen T. L. H., Wang A. (2022). Is There a Gap Between Artificial Intelligence Applications and Priorities in Health Care and Nursing Management?. *Journal of Nursing Management*.

[50] Fuller R., Hansen A. (2019). Disruption Ahead: Navigating and Leading the Future of Nursing. *Nursing Administration Quarterly*.

[51] Cato K. D., McGrow K., Rossetti S. C. (2020). Transforming Clinical Data Into Wisdom: Artificial Intelligence Implications for Nurse Leaders. *Nursing Management*.

[52] Al Kuwaiti A., Nazer K., Al-Reedy A. (2023). A Review of the Role of Artificial Intelligence in Healthcare. *Journal of Personalized Medicine*.

[53] Secinaro S., Calandra D., Secinaro A., Muthurangu V., Biancone P. (2021). The Role of Artificial Intelligence in Healthcare: A Structured Literature Review. *BMC Medical Informatics and Decision Making*.

[54] Yin J., Ngiam K. Y., Teo H. H. (2021). Role of Artificial Intelligence Applications in Real-Life Clinical Practice: Systematic Review. *Journal of Medical Internet Research*.

[55] Shelmerdine S. C., Arthurs O. J., Denniston A., Sebire N. J. (2021). Review of Study Reporting Guidelines for Clinical Studies Using Artificial Intelligence in Healthcare. *BMJ Health & Care Informatics*.

[56] Davenport T. H., Ronanki R., Wheaton J., Nguyen A. (2018). Feature Artificial Intelligence for the Real World. *Harvard Business Review*.

[57] Neill D. B. (2013). Using Artificial Intelligence to Improve Hospital Inpatient Care. *IEEE Intelligent Systems*.

[58] Bughin J., Hazan E., Ramaswamy S. (2017). *Artificial Intelligence the Next Digital Frontier?*.

[59] Ransbotham S., Khodabandeh S., Fehling R., Lafountain B., Kiron D. (2019). *Winning With AI*.

[60] Jobin A., Ienca M., Vayena E. (2019). The Global Landscape of AI Ethics Guidelines. *Nature Machine Intelligence*.

[61] Mittelstadt B. D., Allo P., Taddeo M., Wachter S., Floridi L. (2016). The Ethics of Algorithms: Mapping the Debate. *Big Data & Society*.

[62] Pailaha A. D. (2023). The Impact and Issues of Artificial Intelligence in Nursing Science and Healthcare Settings. *SAGE Open Nursing*.

[63] Lee D., Yoon S. N. (2021). Application of Artificial Intelligence-Based Technologies in the Healthcare Industry: Opportunities and Challenges. *International Journal of Environmental Research and Public Health*.

[64] Tambe P., Cappelli P., Yakubovich V. (2019). Artificial Intelligence in Human Resources Management: Challenges and a Path Forward. *California Management Review*.

[65] Starke C., Baleis J., Keller B., Marcinkowski F. (2022). Fairness Perceptions of Algorithmic Decision-Making: A Systematic Review of the Empirical Literature. *Big Data & Society*.

[66] Baxter S. L., Bass J. S., Sitapati A. M. (2020). Barriers to Implementing an Artificial Intelligence Model for Unplanned Readmissions. *ACI Open*.

[67] Smith T. G., Norasi H., Herbst K. M. (2022). Creating a Practical Transformational Change Management Model for Novel Artificial Intelligence-Enabled Technology Implementation in the Operating Room. *Mayo Clinic Proceedings: Innovations, Quality & Outcomes*.

[68] Reddy S. (2024). Generative AI in Healthcare: An Implementation Science Informed Translational Path on Application, Integration and Governance. *Implementation Science*.

[69] Duncan R., Eden R., Woods L., Wong I., Sullivan C. (2022). Synthesizing Dimensions of Digital Maturity in Hospitals: Systematic Review. *Journal of Medical Internet Research*.

[70] Shrivastav M. (2022). Barriers Related to AI Implementation in Supply Chain Management. *Journal of Global Information Management*.

[71] Liebowitz J. (2001). Knowledge Management and Its Link to Artificial Intelligence. *Expert Systems with Applications*.

