# Supplementary material for: Impact of Artificial Intelligence–Based Technology on Nurse Management: A Systematic Review
Source: J Nurs Manag. 2024 Oct 12;2024:3537964. doi: 10.1155/2024/3537964 (PMC11919197; doi:10.1155/2024/3537964)
Supplement: Supporting Information — File 1: Table S1: article search syntax; Table S2: overview of conceptual synthesis and discussion; Table S3: quality appraisal of quantitative papers; Table S4: quality appraisal of qualitative papers; Table S5: risk of bias assessment for included studies; Table S6: applicability study of articles to key nursing management areas; Table S7: integration of AI in nursing management practice; Table S8: AI barriers and challenges; Table S9: future trends and recommendations for the integration of AI in nursing management. [file 3537964.f1.docx]

**Supplementary file 1**

Table S1. Overview of the included studies

| Authors (year) | Design | Participants (n) and Population | Variable(s) | Type of AI | Application in Nursing | Main Findings | Risk of Bias |
| --- | --- | --- | --- | --- | --- | --- | --- |
| (Han et al., 2020) | Descriptive, cross-sectional survey | 222 experienced nurses from a general hospital | Intelligent healthscape quality, job outcomes, job satisfaction, pleasure feeling, innovativeness | Intelligent healthscape (e.g., smart wards, nursing assistive robot, advanced operating rooms) | Impact on job outcomes and job satisfaction through pleasure feeling | Intelligent healthscape quality positively affected job outcomes and satisfaction through pleasure feeling. Pleasure feeling's effects on outcomes were stronger in nurses with higher levels of innovativeness. | Low |
| (Chang et al., 2022) | Bibliometric analysis | N/A (Not a participant-based study) | Trends and impact of AI in nursing research; Publications and citations; Institutional and country contributions; Research areas and keywords in AI nursing research | Artificial Intelligence as conceptualized in nursing literature | Investigation of academic use of AI in nursing | The study highlights a significant growth in AI research within nursing, emphasizing the contributions of the US and focusing on machine learning and natural language processing. | Low |
| (Chen et al., 2022) | Conceptual analysis | N/A | Gap between AI application developments and nursing management priorities | N/A | Examining the alignment between AI developments and nursing management priorities | Identified a conceptual gap between AI applications and nursing management priorities, emphasizing the need for closer alignment to address nursing management challenges effectively. | Moderate |
| (Ergin et al., 2022) | Descriptive cross-sectional study | 326 nurse managers from 20 university hospitals in Turkey | Nurse managers' opinions on AI and robot nurses | Artificial intelligence and robot nurses | Assessment of nurse managers' perceptions and opinions on the use of AI and robot nurses in healthcare | The majority of nurse managers were aware of AI and robot nurses, with many believing that these technologies would benefit nursing by reducing workload, despite skepticism about robots replacing human nurses. There was a strong emphasis on the need for education and training in AI and robotics within nursing curricula. | High |
| (Huang et al., 2022) | Two-wave study | N/A | Efficiency of nurse-patient interaction and reduction of nurses’ working hours | AI-based intelligent surveillance | Implementation in a nursing home to monitor patient status and assist nurses | AI-based surveillance significantly reduced the average nurse-patient interaction time from 18 to 10 minutes per patient, enhancing nursing efficiency and patient care. However, limitations such as potential privacy concerns and the need for additional devices were noted. | Moderate |
| (Laukka et al., 2022) | Descriptive qualitative study | Nurse leaders (n = 20) and digital service developers (n = 10) | Perceptions of AI's future role in specialized medical care | General AI applications in healthcare | Understanding the future implications of AI in nursing and healthcare management | AI is envisioned to transform work, care, services, and organizational structures in specialized healthcare, serving as a complement to human clinicians rather than a replacement. The study highlights the importance of AI in enhancing efficiency, decision-making, and patient care, with an emphasis on the need for leadership in navigating the integration of AI into healthcare practices. | Moderate |
| (Blouin, 2023) | Conceptual discussion/review | N/A | Integration of technology in nursing workforce management; Reduction of non-value-added time | Various AI and emerging technologies | Enhancing nurse recruitment, engagement, satisfaction, and retention; Addressing nursing workforce shortages through technology; Reducing administrative tasks and non–value-added activities for nurses; Improving the efficiency and quality of nursing care | Emerging technologies, when carefully selected and evaluated, can supplement traditional strategies to address nursing workforce challenges, potentially improving nurse satisfaction, reducing workload, and enhancing patient care quality and safety. | Moderate |
| (Li et al., 2022) | Cross-sectional survey | 263 Chinese nurses from tertiary and secondary hospitals | Leaders’ innovation expectation, nurses’ innovation behavior, job control, creative self-efficacy | Artificial intelligence in healthcare | Investigating the influence of leaders' innovation expectation on nurses' innovation behavior with AI, and the mediating roles of job control and creative self-efficacy | Leaders' innovation expectations positively influence nurses' innovation behavior in AI, mediated by creative self-efficacy and job control. The study highlights the importance of supportive leadership and empowerment in fostering innovation among nurses in the context of AI. | Moderate |
| (Wang et al., 2022) | Cross-sectional study | 511 elderly caregivers in 25 elderly institutions | AI's information quality, system quality, and service quality; Psychological and structural empowerment; Retention intention | AI applications in elderly care | Exploring the influence of AI use on psychological and structural empowerment and its association with the retention of elderly caregivers | The study revealed that AI's information, system, and service quality significantly positively impact both psychological and structural empowerment of elderly caregivers, which in turn, positively affects their retention intentions. The joint explanatory power for retention intention was 42.6%. | Moderate |
| (Fuller & Hansen, 2019) | Conceptual discussion | N/A | Disruptive vs. sustaining innovation; Roles of nurse leaders in navigating healthcare innovation | N/A | The role of nurse leaders in navigating and leading future healthcare innovations | Differentiates between disruptive and sustaining innovations, highlighting the potential for AI, virtual reality, and other technologies to drive significant changes in healthcare delivery and management. It emphasizes the pivotal roles nurse leaders play as influencers, validators, and strategic advisors in fostering a culture of innovation and in steering the nursing profession through upcoming changes. | Moderate |
| (Clancy, 2020b) | Conceptual discussion | N/A | Technology applications in nursing including EHRs, data analytics, robotics, and IoT | Various AI and technology applications | Examines how technology can improve nurse productivity, reduce waste, and enhance healthcare delivery | The article highlights how leveraging technology can significantly improve healthcare outcomes by enhancing nurse productivity, reducing waste through efficiency improvements, and ultimately improving patient care. It emphasizes the role of nurse leaders in adopting and integrating these technologies into healthcare practices. | Moderate |
| (Cato et al., 2020) | Conceptual discussion | N/A | Integration of AI and CDS within nursing practice; Application of the DIKW model in nursing informatics | Various AI technologies and machine learning | Examines the role of AI and CDS in transforming clinical data into wisdom, enhancing nursing practice and decision-making | The article emphasizes the importance of integrating AI with CDS applications to leverage the vast amounts of data available in healthcare. It advocates for the strategic implementation of AI/CDS tools to support nurses in decision-making processes, enhance patient care, and optimize operational efficiency. The DIKW framework is highlighted as a valuable model for understanding and applying AI and CDS in nursing. | Moderate |
| (Clancy, 2020a) | Conceptual discussion | N/A | The integration and potential of AI in healthcare, with a focus on nursing applications | Machine learning, deep learning, voice and facial recognition software, personal virtual assistants, and robotics | Discussion on how AI can improve nursing practice, patient care, and healthcare operations, including voice recognition for clinical documentation, digital phenotyping for disease diagnosis, and autonomous robots for various tasks | The article posits that AI will have a transformative impact on nursing by automating complex tasks, enhancing patient care through advanced predictive models, and potentially improving healthcare efficiency and outcomes. | Moderate |
| (Fontenot, 2024) | Conceptual discussion | N/A | Integration of AI into nursing leadership and healthcare | Various AI technologies | Provides insights on utilizing AI for evidence-based practice in healthcare settings, addressing ethical challenges and strategies for research translation | The article underscores the importance of AI in closing the research-to-practice gap, promoting optimal patient safety, and enhancing care delivery methods. It emphasizes the need for nurse leaders to understand and leverage AI technologies effectively while addressing ethical considerations and ensuring the appropriate use of AI in clinical settings. | Moderate |

Table S2. Overview of Conceptual synthesis and discussion

| Citation | Main Themes | Key Concepts | Central Propositions | Implications for Nursing Management | Reflections on AI |
| --- | --- | --- | --- | --- | --- |
| (Fontenot, 2024) | Future role of AI in nursing | Leadership, change management | Leaders need to embrace AI to advance nursing practice | Encourage proactive leadership in integrating AI technologies | AI as a transformative tool in nursing |
| (Clancy, 2020b) | Technology support in nursing | Productivity, efficiency | Technological tools can enhance nursing productivity | Integrate tech solutions to optimize nursing workflows | Highlighting the value of AI in resource optimization |
| (Clancy, 2020a) | Leadership and AI | Organizational change, AI integration | Nursing leaders play a critical role in AI adoption | Develop leadership skills focused on technology management | AI as an opportunity for nursing leadership to drive innovation |
| (Blouin, 2023) | AI in workforce management | Workforce optimization, administrative efficiency | AI can reduce administrative tasks for nurses | Adopt AI to improve nursing workforce management and patient care | Balancing technology and human aspects in nursing care |
| (Chen et al., 2022) | AI's gap in nursing priorities | Conceptual gap, management priorities | Identifying the disconnect between AI development and nursing management needs | Align AI development with the strategic goals of nursing management | Need for strategic alignment between AI capabilities and nursing needs |
| (Laukka et al., 2022) | AI's impact on specialized medical care | Decision-making enhancement, efficiency | AI is seen as an adjunct to human decision-making in healthcare | Train nursing leaders on the potential and limitations of AI | Emphasizing AI's role in supporting, not replacing, clinical judgement |
| (Fuller & Hansen, 2019) | Integration of AI in nursing | Process improvement, predictive analysis | AI improves patient care quality and efficiency | Foster collaboration between nursing staff and AI developers | A proactive approach towards technological advancements in healthcare |
| (Cato et al., 2020)) | DIKW framework and AI applications | DIKW framework, clinical decision support | AI enhances clinical decision-making through the DIKW framework | Educate nursing staff on AI and its applications in clinical settings | The importance of understanding AI's role and limitations |

Table S3. Quality Aprraisal of Quantitative papers

| Citations | Q1 | Q2 | Q3 | Q4 | Q5 | Q6 | Q7 | Q8 | Total score | Share of answers yes (%) |
| --- | --- | --- | --- | --- | --- | --- | --- | --- | --- | --- |
| (Han et al., 2020) | Yes | Yes | Yes | Yes | Unclear | Unclear | Yes | Yes | 6/8 | 75% |
| (Chang et al., 2022) | Yes | Yes | N/A | Yes | No | No | Yes | Yes | 5/8 | 62.5% |
| (Wang et al., 2022) | Yes | Yes | N/A | Yes | No | No | Yes | Yes | 5/8 | 62.5% |
| (Huang et al., 2022) | Yes | Yes | N/A | Yes | No | No | Yes | Yes | 5/8 | 62.5% |
| (Ergin et al., 2022) | Yes | Yes | No | No | No | No | Yes | Yes | 4/8 | 50% |
| (Li et al., 2022) | Yes | Yes | N/A | No | No | No | Yes | Yes | 4/8 | 50% |

Table S4. Quality Aprraisal of qualitative papers

| Citations | Q1 | Q2 | Q3 | Q4 | Q5 | Q6 | Q7 | Q8 | Q9 | Q10 | Total score | Share of answers yes (%) |
| --- | --- | --- | --- | --- | --- | --- | --- | --- | --- | --- | --- | --- |
| (Fontenot, 2024) | N/A | N/A | N/A | N/A | N/A | Unclear | Unclear | N/A | N/A | Yes | 1/10 | 10% |
| (Clancy, 2020a) | N/A | Yes | N/A | N/A | Yes | No | No | N/A | N/A | Yes | 3/10 | 30% |
| (Clancy, 2020a) | N/A | Yes | N/A | N/A | Yes | No | No | N/A | N/A | Yes | 3/10 | 30% |
| (Blouin, 2023) | N/A | Yes | N/A | N/A | Yes | No | No | N/A | N/A | Yes | 3/10 | 30% |
| (Chen et al., 2022) [49] | N/A | Yes | N/A | N/A | Yes | No | No | N/A | N/A | Yes | 3/10 | 30% |
| (Laukka et al., 2022) | Yes | Yes | Yes | Yes | Yes | N/A | N/A | Yes | Yes | Yes | 8/10 | 80% |
| (Fuller & Hansen, 2019) | N/A | Yes | N/A | N/A | Yes | No | No | N/A | N/A | Yes | 3/10 | 30% |
| (Cato et al., 2020) | N/A | Yes | N/A | N/A | Yes | No | No | N/A | N/A | Yes | 3/10 | 30% |

Table S5. Risk of Bias Assessment for Included Studies

| Author (Year) | Study Design | Sample Size | Bias Evaluation | Risk of Bias |
| --- | --- | --- | --- | --- |
| Han et al. (2020) | Descriptive cross-sectional survey | 222 | High methodological quality in sampling and data analysis | Low |
| Chang et al. (2022) | Bibliometric analysis | N/A | Robust and comprehensive literature evaluation | Low |
| Chen et al. (2022) | Conceptual analysis | N/A | Conceptual evaluation without empirical data, possible theoretical limitations | Moderate |
| Blouin (2023) | Conceptual discussion | N/A | Well-founded theoretical analysis but lacks empirical data | Moderate |
| Ergin et al. (2022) | Descriptive cross-sectional study | 326 | Sampling and analysis methods with possible biases | High |
| Huang et al. (2022) | Two-wave study | N/A | Reduction in working hours, privacy limitations | Moderate |
| Laukka et al. (2022) | Descriptive qualitative study | 30 | Good practices in interviews and thematic analysis, sample size limitations | Moderate |
| Li et al. (2022) | Cross-sectional survey | 263 | Solid evaluation with some limitations in variable control | Moderate |
| Wang et al. (2022) | Cross-sectional survey | 382 | Adequate methods with some data analysis limitations | Moderate |
| Fuller & Hansen (2019) | Conceptual discussion | N/A | Well-founded theoretical analysis without empirical data | Moderate |
| Clancy (2020b) | Conceptual discussion | N/A | Theoretical evaluation of technological solutions for nursing leaders, no empirical data | Moderate |
| Cato et al. (2020) | Conceptual discussion | N/A | Theoretical evaluation of DIKW framework applied to AI and clinical decision support in nursing | Moderate |
| Clancy (2020a) | Conceptual discussion | N/A | Theoretical evaluation of AI in nursing and its potential impact | Moderate |
| Fontenot (2024) | Conceptual discussion | N/A | Theoretical analysis of AI use in evidence-based practice and nursing management | Moderate |

Table S6. Applicability Study of Articles to Key Nursing Management Areas

| Author/Year | Nursing Management Areas | Yes/No | Reason if Applicable |
| --- | --- | --- | --- |
| (Blouin, 2023) | Decision Making | Yes | Blouin discusses the integration of emerging technologies alongside traditional strategies to address the nursing workforce shortage, specifically highlighting how technology supports decision-making to optimize engagement, capacity, and efficiency. |
|  | Leadership | Yes | Leadership is emphasized in technology adoption. |
|  | Human Resources Management | Yes | The article explores human resource management by suggesting technology can reduce non-value-added activities for nurses, thereby improving satisfaction and retention. |
|  | Material Resources Management | Yes | Material resource management is explored with technology helping to reduce non-value-added activities. |
|  | Communication | Yes | Focuses on improving communication through technological solutions. |
|  | Ethics and Values | Yes | Emphasizes ethical considerations in implementing AI. |
|  | Team Management | Yes | The integration of emerging technologies affects multiple management areas, including team management. |
|  | Conflict Management | No | Conflict management isn't directly addressed in the context of technology integration. |
| (Han et al., 2020) | Decision Making | Yes | Innovative technology enhances decision-making and leadership by providing real-time monitoring and support for healthcare decisions. |
|  | Leadership | Yes | Leadership abilities are enhanced by real-time monitoring and support for healthcare decisions. |
|  | Human Resources Management | Yes | Effective human resources management is facilitated by innovative technology. |
|  | Material Resources Management | Yes | Materials management is facilitated by innovative technology in the healthcare environment. |
|  | Communication | Yes | Enhanced communication technologies contribute to better team coordination. |
|  | Ethics and Values | Yes | Ethical considerations in care delivery are supported by enhanced communication technologies. |
|  | Team Management | Yes | Team management benefits from the presence of innovative technologies improving coordination. |
|  | Conflict Management | No | Conflict management wasn't directly addressed. |
| (Chang et al., 2022) | Decision Making | Yes | AI supports decision-making processes, promotes leadership development, and improves management through training programs and healthcare delivery optimization. |
|  | Leadership | Yes | Leadership development is promoted through AI technologies. |
|  | Human Resources Management | Yes | Human resources management is improved through AI-enabled training programs. |
|  | Material Resources Management | Yes | Material resource management is enhanced by optimizing healthcare delivery through AI technologies. |
|  | Communication | Yes | Communication is fostered through AI-enabled tools. |
|  | Ethics and Values | Yes | Ethical considerations in AI deployment are crucial, emphasizing the integration of AI ethically and responsibly. |
|  | Team Management | Yes | Team management benefits from AI's ability to streamline operations and improve efficiency. |
|  | Conflict Management | No | Direct implications for conflict management were not explicitly addressed. |
| (Chen et al., 2022) | Decision Making | Yes | Discusses the misalignment between AI developments and nursing management priorities, impacting decision making and leadership. |
|  | Leadership | Yes | Leadership in bridging AI technology with nursing care practices is emphasized. |
|  | Human Resources Management | No | Does not directly address human resources management. |
|  | Material Resources Management | No | Does not directly address material resources management. |
|  | Communication | Yes | Highlights the need for better communication between AI developers and nursing managers. |
|  | Ethics and Values | Yes | Emphasizes the need to ensure AI technologies align with nursing's ethical standards. |
|  | Team Management | No | Does not directly address team management. |
|  | Conflict Management | No | Does not directly address conflict management. |
| (Ergin et al., 2022) | Decision Making | Yes | AI supports decision-making processes by providing timely, accurate information and assistive functionalities in healthcare settings. |
|  | Leadership | Yes | Leadership in integrating AI technologies into nursing practice is highlighted, ensuring ethical use. |
|  | Human Resources Management | Yes | AI and robot nurses can complement human resources by reducing nurses' workload. |
|  | Material Resources Management | No | Does not directly address material resources management in detail. |
|  | Communication | Yes | Effective communication between healthcare professionals and AI systems is fostered. |
|  | Ethics and Values | Yes | The study underscores the significance of ethical considerations in AI use. |
|  | Team Management | Yes | AI's role in supporting team management through enhanced informational and assistive functionalities is suggested. |
|  | Conflict Management | No | Conflict management is not directly addressed. |
| (Huang et al., 2022) | Decision Making | Yes | AI facilitates decision-making by providing nurses with statistical health data to intuitively grasp patients' statuses. |
|  | Leadership | Yes | Leadership is highlighted in the integration and ethical use of AI technologies. |
|  | Human Resources Management | Yes | The effectiveness of AI-based intelligent surveillance reduces nurses' working hours, supporting human resources management. |
|  | Material Resources Management | No | Does not directly address material resources management. |
|  | Communication | Yes | Communication improvements are noted as AI provides real-time responses to patients' abnormal health statuses. |
|  | Ethics and Values | Yes | Ethical considerations are addressed in the use of AI to support nursing care while ensuring patient privacy and dignity. |
|  | Team Management | Yes | Team management benefits from AI's assistance in streamlining nurse-patient interactions. |
|  | Conflict Management | No | Conflict management was not explicitly discussed. |
| (Laukka et al., 2022) | Decision Making | Yes | AI significantly influences future specialized helathcare, supporting decision-making through enhanced data analysis and prediction capabilities. |
|  | Leadership | Yes | Leadership is essential in guiding the integration of AI technologies, with a focus on developing AI literacy among nursing staff. |
|  | Human Resources Management | Yes | AI literacy among nursing staff is crucial for managing human resources effectively. |
|  | Material Resources Management | No | Material resource management wasn't directly discussed. |
|  | Communication | Yes | Communication improvements through AI technologies facilitate better patient engagement and care coordination. |
|  | Ethics and Values | Yes | Ethical considerations are crucial in ensuring AI is implemented responsibly, aligning with healthcare values and standards. |
|  | Team Management | Yes | Team management benefits from AI by optimizing workflows and task allocations. |
|  | Conflict Management | No | Conflict management through AI was not a focus of the study. |
| (Li et al., 2022) | Decision Making | Yes | The study underscores the importance of leaders' innovation expectations in fostering an environment that encourages nurses to innovate within the context of artificial intelligence (AI), directly relating to decision-making as it empowers nurses with autonomy and self-efficacy to make innovative decisions. |
|  | Leadership | Yes | Leadership is crucial in setting innovation expectations, influencing how nurses manage human resources by promoting a culture of innovation and creativity. |
|  | Human Resources Management | Yes | Influences human resources by promoting a culture of innovation and creativity among nurses. |
|  | Material Resources Management | No | The study does not directly address material resources management. |
|  | Communication | Yes | Communication is enhanced through the shared understanding and pursuit of innovation goals. |
|  | Ethics and Values | No | Ethics and values are not directly discussed in the context of AI application in nursing. |
|  | Team Management | No | Does not specifically address conflict management. |
|  | Conflict Management | No | Does not address conflict management. |
| (Wang et al., 2022) | Decision Making | Yes | AI's role in enhancing empowerment affects decision-making by enabling caregivers to make more informed decisions regarding care. |
|  | Leadership | Yes | Leadership is highlighted as crucial for fostering an AI-enabled environment that supports caregiver empowerment and retention. |
|  | Human Resources Management | Yes | AI empowerment (psychological and structural) leads to higher retention intentions among caregivers, effectively managing human resources. |
|  | Material Resources Management | No | Does not specifically address material resources management. |
|  | Communication | Yes | Communication benefits from AI's quality, facilitating better interaction and support. |
|  | Ethics and Values | No | Ethics and values are not directly discussed, but the emphasis on quality AI use suggests an underlying concern for ethical implications in caregiving. |
|  | Team Management | No | Does not specifically address conflict management. |
|  | Conflict Management | No | Does not specifically address conflict management. |
| (Fuller & Hansen, 2019) | Decision Making | Yes | Discusses disruptive innovations and their impact, asserting that nurse leaders must make informed decisions on adopting new technologies. |
|  | Leadership | Yes | Nurse leaders are described as influencers, validators, and strategic advisors essential for integrating innovations like AI and VR into healthcare. |
|  | Human Resources Management | Yes | Managing human resources by preparing the nursing workforce for future challenges is emphasized. |
|  | Material Resources Management | No | Material resources management is not directly addressed. |
|  | Communication | Yes | Communicating effectively about innovations and their implications is crucial. |
|  | Ethics and Values | Yes | Ethical considerations in integrating innovations responsibly to improve patient care are highlighted. |
|  | Team Management | Yes | Nurse leaders are seen as key to leading teams through changes introduced by disruptive innovations. |
|  | Conflict Management | No | Conflict management is not directly addressed. |
| (Clancy, 2020b) | Decision Making | Yes | Clancy discusses the vital role of technology in making informed decisions and leading effectively by integrating technology into healthcare. |
|  | Leadership | Yes | Emphasizes nurse leaders' role in adopting technology for productivity and quality improvements. |
|  | Human Resources Management | Yes | Manages human resources by optimizing nurse productivity through technology. |
|  | Material Resources Management | Yes | Manages material resources through technology-enhanced supply chain management. |
|  | Communication | Yes | The importance of communication technologies in improving information flow and coordination is underscored. |
|  | Ethics and Values | Yes | Acknowledges the ethical considerations in the use of technology in nursing, particularly regarding patient data and privacy concerns. |
|  | Team Management | Yes | Focuses on using technology to enhance team management and collaboration among healthcare professionals. |
|  | Conflict Management | No | Although conflict management is not directly addressed, the focus is on using technology to enhance collaboration. |
| (Cato et al., 2020) | Decision Making | Yes | Explores the significance of the DIKW framework, focusing on how AI and CDS systems transform clinical data into wisdom, enhancing decision-making by providing nurses with actionable insights and supporting evidence-based practice. |
|  | Leadership | Yes | Emphasizes leadership in the strategic implementation and ethical considerations of AI/CDS tools, ensuring technology augments rather than replaces human judgment. |
|  | Human Resources Management | No | Does not directly address human resources management. |
|  | Material Resources Management | No | Does not directly address material resources management. |
|  | Communication | Yes | Communication between healthcare providers is facilitated by AI/CDS, improving the clarity and efficiency of clinical information exchange. |
|  | Ethics and Values | Yes | Discusses the ethical application of AI in healthcare, highlighting the importance of transparency and accountability in AI systems to support patient care. |
|  | Team Management | No | Does not directly address team management. |
|  | Conflict Management | No | Does not directly address conflict management. |
| (Clancy, 2020a) | Decision Making | Yes | Highlights how AI can support decision-making through advanced data analysis and learning capabilities. |
|  | Leadership | Yes | Underlines leadership's role in guiding the integration of AI into nursing practices, including navigating ethical considerations. |
|  | Human Resources Management | Yes | Points to human resources management by improving nursing workflows and patient care through AI-enhanced tools. |
|  | Material Resources Management | Yes | Material resources management is addressed through AI's potential to optimize supply chain and resource allocation. |
|  | Communication | Yes | Envisions communication improvements with voice and facial recognition technologies, enhancing interactions between healthcare providers and patients. |
|  | Ethics and Values | Yes | Ethics and values are central, considering the implications of AI on patient privacy and data security. |
|  | Team Management | Yes | Team management benefits from AI's ability to provide comprehensive patient data, fostering collaboration among healthcare professionals. |
|  | Conflict Management | No | Conflict management isn't directly discussed, but the overarching themes suggest AI's role in mitigating challenges through better information and resource management. |
| (Fontenot, 2024) | Decision Making | Yes | Discusses AI's role in expediting evidence-based practice and improving patient outcomes, automating and enhancing decision-making processes. |
|  | Leadership | Yes | AI impacts nursing leadership's strategic planning and operational efficiency. |
|  | Human Resources Management | Yes | Highlights AI's facilitation of human resource management through the optimization of nursing workflows. |
|  | Material Resources Management | Yes | Indirectly addresses material resource management by improving the allocation of healthcare resources. |
|  | Communication | Yes | Points to significant enhancement of communication through AI tools that can analyze and disseminate complex healthcare data efficiently. |
|  | Ethics and Values | Yes | Ethical challenges surrounding AI in clinical settings are addressed, stressing the importance of responsible AI use that aligns with nursing ethics and values. |
|  | Team Management | Yes | Underlines the necessity for nurse leaders to understand and navigate challenges, effectively managing teams in the context of AI integration. |
|  | Conflict Management | Yes | Discusses managing conflicts that may arise from the implementation of AI technologies in healthcare practices. |

1. Decision Making: Indica si el artículo proporciona información relevante para la toma de decisiones en la gestión de enfermería.

2. Leadership: Evalúa si el artículo aborda aspectos del liderazgo en el contexto de la gestión de enfermería.

3. Human Resources Management: Considera si el artículo trata temas relacionados con la gestión de recursos humanos.

4. Material Resources Management: Identifica si el artículo incluye discusiones sobre la gestión de recursos materiales.

5. Communication: Verifica si el artículo examina la importancia de la comunicación dentro de la gestión de enfermería.

6. Ethics and Values: Evalúa si el artículo considera la ética y los valores en su discusión sobre IA en enfermería.

7. Team Management: Determina si el artículo aborda la gestión de equipos dentro del contexto de enfermería.

8. Conflict Management: Comprueba si el artículo incluye elementos relacionados con la gestión del conflicto.

Table S7. Integration of AI in nursing management practice

| Citation | AI Applications | Impact on Practice |
| --- | --- | --- |
| (Blouin, 2023) | Integration of emerging technologies to manage the nursing workforce shortage. | Improvement in nurse recruitment, retention, and satisfaction; reduction of administrative tasks. |
| (Han et al., 2020) | The study examined the influence of intelligent healthscape quality (IHQ) on nurse job outcomes and satisfaction. IHQ encompasses the integration of advanced technology, safety features, ambiance, layout accessibility, and sociality within the healthcare setting, focusing on AI and medical robotics (e.g., Da Vinci™, Watson™, TUG™) application along with Information and Communications Technologies (ICTs) to enhance the nursing work environment. | Findings indicated that a high quality of intelligent healthscape significantly impacts nurses' job outcomes and satisfaction by fostering a positive work environment. The application of AI and intelligent systems in healthcare settings was shown to increase job satisfaction and outcomes through improved working conditions, enabling nurses to concentrate more effectively on patient care and reducing administrative burdens. |
| (Chang et al., 2022) | Investigation of the academic use of AI in nursing through a bibliometric analysis, emphasizing AI's role in promoting healthcare quality and nursing staff's AI literacy. | Highlights the potential of AI to improve nursing management, quality, safety management, and team communication, and encourages future international collaboration. |
| (Chen et al., 2022) | Evaluates the alignment between AI applications and nursing management priorities, highlighting a potential gap in addressing managerial needs through technology. | Suggests a reevaluation of AI focus to ensure it aligns with critical nursing management priorities such as staff retention, practice and quality of care, and financial responsibility. Calls for closer collaboration between AI developers and nursing management to bridge this gap. |
| (Ergin et al., 2022) | The study explores nurse managers' opinions on AI and robot nurses, indicating a growing awareness and acceptance among nursing leadership. It examines their beliefs on the benefits and limitations of such technologies in healthcare. | The majority of nurse managers foresee AI and robot nurses as beneficial adjuncts that won't replace human nurses but will augment the nursing practice by reducing workload and potentially improving patient care efficiency and safety. |
| (Huang et al., 2022) | Developed an AI-based intelligent surveillance system to monitor patients’ health status and generate statistical health data, aimed at reducing nurses’ working hours in nurse-patient interaction. | Reduced the average nurse-patient interaction time per patient from 18 to 10 minutes, improving nursing efficiency by 42.95%. AI’s real-time response to patient health status abnormalities also enhances emergency detection and intervention. |
| (Laukka et al., 2022) | Explored nurse leaders' and digital service developers' perceptions on the future role of AI in specialized healthcare, focusing on its transformative potential in work, care, services, and organizational structures. | Predicted a significant role for AI in specialized healthcare, emphasizing its ability to supplement rather than replace human clinicians. AI is expected to free up clinicians' time, support personalized patient care, enhance decision-making, and improve organizational efficiency and safety. |
| (Clancy, 2020b) | Clancy discusses AI's broad applications in nursing, including voice and facial recognition software for clinical documentation, personal virtual assistants for data integration and patient care recommendations, and robotics for various assistive tasks. | Predicts a transformational impact on nursing, automating routine tasks to focus more on patient care, enhancing data-driven decision-making, and introducing efficiencies in clinical documentation and patient monitoring. |
| (Cato et al., 2020) | Discusses AI in part as computer science and cognitive science, focusing on its integration with clinical models, critical-thinking skills, and evidence-based practice. Highlights include the application of machine learning algorithms that learn from data without human direction, and the use of AI in predictive analytics, personal virtual assistants for data integration, and robotics for assistive tasks in nursing. | The article suggests that AI could significantly enhance nursing practice by automating routine tasks to allow more focus on patient care, supporting data-driven decision-making, and introducing efficiencies in clinical documentation and patient monitoring. The DIKW (Data, Information, Knowledge, Wisdom) framework is used to illustrate how AI and Clinical Decision Support (CDS) systems can advance nursing practice from data processing to wisdom generation. |
| (Li et al., 2022) | Investigation of the influence of leaders’ innovation expectation on nurses’ innovation behavior in conjunction with AI, alongside the mediating effects of job control and creative self-efficacy. | Leaders' innovation expectations were found to positively influence nurses' innovation behavior, enhancing their enthusiasm for innovation. The study suggests that by formulating intervention measures to increase leaders’ innovation expectation, nurses’ creative self-efficacy, and job control, it could encourage nurses’ innovation behavior, thereby potentially improving hospital management efficiency and fostering a culture of continuous improvement and adaptation in the use of medical AI technologies. |
| (Clancy, 2020a) | Utilization of a wide range of technologies including EHRs, data analytics, predictive modeling, AI, ASR, NLP, robotics, and IoT. | Assists in making nurses more productive by reducing waste through technology, contributing to quality, access, and cost improvements. Offers solutions to the global shortage of healthcare workers by enhancing nurse productivity through technological advancements. |
| (Fontenot, 2024) | The article discusses AI's implications in evidence-based practice (EBP), highlighting the use of large language models (LLMs) for automating tasks that can accelerate the translation of research into clinical practice. Strategies include utilizing AI tools like ChatGPT for developing search strategies and Elicit for managing the synthesis process of systematic searches. | AI is posited to significantly speed up EBP in clinical settings, reducing the traditional gap between research and practice implementation. This hastened translation can promote optimal patient safety and care delivery methods. The article emphasizes the need for nurse leaders to be aware of and engage with AI tools to facilitate this process while also considering the ethical challenges of using AI in clinical settings. |
| (Wang et al., 2022) | Investigates the association between AI use and the retention of elderly caregivers, focusing on how AI supports caregivers in daily services and reduces work pressure. | Found that AI quality positively affects caregiver empowerment, leading to higher retention intentions. Highlights the need for elderly care institutions to improve AI quality and consider AI's role in structural empowerment to enhance caregiver retention through education, training, and resource support. |
| (Fuller & Hansen, 2019) | Discussion on the concepts of innovation and disruptive innovation within healthcare, highlighting the emergence of artificial intelligence (AI), virtual reality (VR), and other technologies as potential disruptors in healthcare. Focuses on the roles of nurse leaders in navigating these disruptions. | Emphasizes the critical roles of nurse leaders as influencers, validators, and strategic advisors in introducing and implementing AI and other disruptive innovations to improve healthcare outcomes. Highlights the importance of nurse leaders in ensuring these technologies align with patient safety, quality, and affordability of healthcare. |

Table S8. AI barriers and challenges

| Citation | Challenges Identified | Barriers Identified |
| --- | --- | --- |
| (Blouin, 2023) | 1. Nursing shortages exacerbated by the pandemic. | 1. Resistance to change due to increased reliance on agency nurses. |
|  | 2. Increased turnover and retirement. | 2. Difficulty in integrating AI with current workflows. |
|  | 3. Fatigue and disillusionment among nurses. | 3. Cost and complexity of implementing new technologies like EHRs. |
|  | 4. High administrative workload. | 4. Lack of interoperability and effective data transfer across different health systems. |
| (Han et al., 2020) | 1. Need for adaptation of nurses to new workflows involving AI and robotics. | 1. Lack of training and familiarity with advanced technologies among nursing staff. |
|  | 2. Potential reduction in human-centric skills due to over-reliance on technology. | 2. Resistance to shifting from traditional to tech-driven processes. |
|  | 3. Variability in acceptance and effective use of technology based on individual innovativeness. | 3. High initial costs and maintenance expenses of implementing AI technologies in healthcare. |
| (Ergin et al., 2022) | 1. Belief that AI and robot nurses will not replace human nurses but assist in reducing workload. | 1. Ethical concerns regarding responsibility for care outcomes when AI is involved. |
|  | 2. Concerns over the ethical implications and the relational aspects of care that AI and robots | 2. Resistance from nursing staff due to fears of job displacement and over-reliance on technology. |
|  | might not fulfill adequately. | 3. Technological and training limitations in fully adopting AI and robot technologies in nursing practice. |
| (Chang et al., 2022) | 1. Resistance to change from nursing staff. | 1. Lack of AI training for nursing staff. |
|  | 2. High initial cost of AI integration. | 2. Concerns about patient data privacy and security. |
| (Chen et al., 2022) | 1. Mismatch between AI applications and actual needs in nursing management. | 1. Lack of collaboration between AI developers and nursing management. |
|  | 2. Insufficient integration of AI into daily nursing workflows. | 2. Insufficient training on AI applications for nursing staff. |
| (Huang et al., 2022) | 1. Limitations of AI in preventing falls and detecting rapid changes in health conditions. | 1. Privacy concerns with AI surveillance, reliance on additional devices for AI implementation. |
| (Laukka et al., 2022) | 1. Integration of AI within existing healthcare systems. | 1. Resistance from healthcare professionals due to lack of understanding and fear of job displacement. |
|  | 2. Addressing ethical concerns related to patient data and privacy. | 2. Technological and infrastructural limitations in current healthcare settings. |
|  | 3. Managing the transformation of work roles and responsibilities. | 3. Need for significant training and development for effective AI adoption. |
| (Li et al., 2022) | 1. Balancing nurses' roles as users and innovators of AI. | 1. Resistance due to fear of job displacement by AI. |
|  | 2. Managing the uncertainty associated with AI technology. | 2. Lack of nurse involvement in AI development. |
|  |  | 3. High demands on nurses' job control and creative self-efficacy. |
| (Wang et al., 2022) | 1. High turnover rates of elderly caregivers exacerbated by poor working conditions. | 1. Inadequate training for caregivers on AI use. |
|  | 2. Lack of professional competence leading to dissatisfaction. | 2. Economic constraints limiting AI integration. |
|  | 3. Risk of AI reducing personal interaction and care quality. | 3. Resistance to technology adoption due to fear of job displacement. |
| (Fuller & Hansen, 2019) | 1. Navigating the transformation of healthcare through disruptive innovation. | 1. Resistance within healthcare systems to adopt new models that integrate AI. |
|  | 2. Ensuring that innovations such as AI enhance rather than disrupt patient care quality. | 2. The risk of increasing costs with the adoption of new technologies. |
| (Clancy, 2020b) | 1. Staffing shortages due to demographic shifts and a retiring workforce. | 1. Inadequate flow of information and poor communication due to poorly designed EHR workflow. |
|  | 2. Performance challenges in health care compared to other sectors. | 2. Resistance to adopting new technologies among healthcare providers. |
| (Cato et al., 2020) | 1. Integration of AI within existing clinical workflows. | 1. Resistance from nursing staff unfamiliar with AI technology. |
|  | 2. Educating nursing staff on AI and CDS capabilities. | 2. Data privacy and security concerns. |
|  | 3. Ensuring AI enhances rather than disrupts patient care. | 3. Cost implications of implementing AI in healthcare settings. |
| (Clancy, 2020a) | 1. Integrating AI into existing complex healthcare systems while maintaining care quality. | 1. Resistance from healthcare professionals due to concerns over AI replacing human judgment and decision-making. |
|  | 2. Overcoming the historical cyclicality of AI interest and funding. | 2. Ethical concerns around data privacy and the use of AI in sensitive medical decisions. |
| (Fontenot, 2024) | 1. Speeding up evidence-based practice while maintaining accuracy and reliability in clinical practice. | 1. Resistance to change and adaptation of new technologies among nursing staff. |
|  | 2. Managing ethical concerns associated with AI use in healthcare. | 2. Lack of infrastructure and resources to fully integrate AI tools like ChatGPT and Elicit into clinical workflows. |
|  |  | 3. Privacy concerns with AI tools that require user data to function. |

Table S9. Future Trends and recommendations for the Integration of AI in Nursing Management

| Citation | Future Trends | Recommendations |
| --- | --- | --- |
| (Blouin, 2023) | Continuous integration of AI to address nursing shortages and enhance operational efficiencies. | Carefully select and evaluate emerging technologies to ensure they meet the practical needs of nursing workflows and enhance patient care quality. |
| (Han et al., 2020) | Growing utilization of AI to enhance the physical and psychological healthscape of nursing work environments. | Emphasize the development and integration of AI-enhanced environments that support nurse well-being and performance, thereby indirectly benefiting patient care quality. |
| (Chang et al., 2022) | The study anticipates increased adoption and integration of AI across various nursing practices. | Encourage further research and cross-domain collaboration to enhance the implementation and effectiveness of AI in nursing. |
| (Chen et al., 2022) | Expectations for the future include a closer alignment of AI tools with the actual needs of nursing management. | Increase collaboration between AI developers and nursing professionals to ensure that future AI applications are more closely aligned with the needs of clinical and management practice in nursing. |
| (Ergin et al., 2022) | Increased integration of AI in daily nursing tasks. | Develop training and ethical guidelines for AI use in nursing. |
| (Huang et al., 2022) | Continued integration of AI to alleviate staffing shortages and improve care efficiency. | Embrace AI technologies while addressing ethical and privacy concerns, and enhance training for nursing staff on new AI tools. |
| (Laukka et al., 2022) | Continued expansion of AI in nursing practices. | Develop comprehensive training programs for nurses to ensure effective and ethical use of AI technologies. |
| (Li et al., 2022) | Increasing influence of AI on nursing management and patient care practices. | Encourage nurse leaders to actively participate in AI training and integration strategies to enhance team effectiveness and patient care. |
| (Wang et al., 2022) | AI will continue to play a critical role in retaining elderly caregivers and improving the efficiency of care. | Focus on enhancing AI service quality and ensuring that caregivers are trained and supported to use AI effectively in their roles. |
| (Fuller & Hansen, 2019) | The trend towards integrating AI in healthcare is set to continue, with a focus on enhancing decision-making processes and operational efficiencies. | Actively participate in strategic planning and training programs to embrace AI technologies effectively, ensuring ethical considerations are addressed. |
| (Clancy, 2020b) | Increasing use of AI to improve health system efficiency and care. | Invest in AI to enhance productivity, especially in predictive models and robotics, to handle routine tasks. |
| (Cato et al., 2020) | Continued expansion of AI in patient monitoring and diagnostics. | Incorporate AI literacy into nursing education to prepare nurses for future technological integrations. |
| (Clancy, 2020a) | Continued advancement and integration of AI in nursing, focusing on enhancing diagnostic tools and patient management systems. | Prioritize AI literacy and provide ongoing education and training to ensure all nursing staff can effectively utilize AI technologies. |
| (Fontenot, 2024) | Continued advancement in AI technologies will further close the research-to-practice gap in nursing. | Ensure the ethical application of AI and foster a culture of continuous learning to keep pace with technological advancements. |
